# Antibacterial efficacy of synthesized silver nanoparticles of *Microbacterium proteolyticum* LA2(R) and *Streptomyces rochei* LA2(O) against biofilm forming meningitis causing microbes

**DOI:** 10.1038/s41598-023-30215-9

**Published:** 2023-03-13

**Authors:** Naushin Bano, Danish Iqbal, Ayoub Al Othaim, Mehnaz Kamal, Hind Muteb Albadrani, Naseh A. Algehainy, Hadeel Alyenbaawi, Fayez Alghofaili, Mohammad Amir, Roohi

**Affiliations:** 1 Protein Research Laboratory, Department of Bioengineering, Integral University, Lucknow, 226026 India; 2 Department of Health Information Management, College of Applied Medical Sciences, Buraydah Private Colleges, Buraydah, 51418 Saudi Arabia; 3 Department of Medical Laboratory Sciences, College of Applied Medical Science, Majmaah University, Al-Majmaah, 11952 Saudi Arabia; 4 Department of Pharmaceutical Chemistry, College of Pharmacy, Prince Sattam Bin Abdulaziz University, Al-Kharj, 11942 Saudi Arabia; 5 Department of Medical Laboratory Technology, Faculty of Applied Medical Sciences, University of Tabuk, Tabuk, 71491 Saudi Arabia

**Keywords:** Microbiology, Medical research

## Abstract

Actinobacteria obtained from the least explored Indian regions were studied for their ability to suppress meningitis-causing bacteria in nanoparticle form. Drug-resistant bacteria and long-term treatment with different medications make meningitis control complicated. Thus, new meningitis drugs are required to combat MDR bacteria. In this study, secondary metabolites isolated from actinomycetes strains, *Microbacterium proteolyticum* LA2(R) and *Streptomyces rochei* LA2(O), were employed to synthesize silver nanoparticles (AgNPs) at 37 °C for seven days incubation. UV–Vis spectroscopy, TEM, FTIR, and HPLC studies were used for the confirmation of the synthesis of AgNPs. Furthermore, these NPs demonstrated antibacterial and antibiofilm activities against meningitis-causing bacteria. The average size of LA2(R) and LA2(O) isolated secondary metabolites mediated AgNPs was observed to be 27 ± 1and 29 ± 2 nm by TEM analysis. FTIR study of RAgNPs and OAgNPs revealed that presence of peaks with positions of 1637.17 cm^1^ and 1636.10 cm^1^ for C=O amide group appearances in the amide I linkage. These NPs were effective against bacterial pathogens such as *S. pneumoniae, H. influenzae,* and *N. meningitidis* and confirmed by their MICs, i.e., 109.4, 120.60, and 138.80 μg/ml of RAgNPs and 105.80, 114.40 and 129.06 μg/ml of OAgNPs, respectively. Additionally, the production of biofilms is impeded by these nanoparticles on *S. pneumoniae*, *H. influenzae*, and *N. meningitidis* by 73.14%, 71.89% and 64.81%, respectively. These findings confirm the potential role of synthesized AgNPs against biofilm forming meningitis causing Multidrug resistance (MDR) microbes.

## Introduction

The term "pathogenic bacteria" refers to bacteria with the potential to cause illness. Bacteria harmful to humans are the main concern for this research work. Pathogenic bacteria can invade common body areas, such as the blood or mucus. Many pathogens can travel through the body's lymphatic and circulatory systems and infiltrate the epithelium, epidermis, or mucous membranes^1^. Specifically, we focus on highly pathogenic bacteria that cause meningitis.

Bacterial meningitis, an infectious syndrome caused by inflammation and infection of the meninges, certainly contributes to global morbidity and mortality^2^. Bacterial meningitis is caused by pathogens *N. meningitidis, H. influenzae,* and *S. pneumoniae*. Bacterial meningitis can be devastating for patients and can occasionally result in death within a few hours. Regardless of whether the patient survives, some hearing and learning problems may continue for a lifetime. The seriousness of this disease can also be imagined in Rizvi et al.'s research report (2018). Patients with bacterial meningitis have a poor prognosis for mortality, and if they do survive, they may develop severe hearing, learning, and memory impairments^3^. The bacterial meningitis-causing bacteria are always pathogenic by nature but also become more dangerous when they form biofilms. Biofilm-embedded bacteria (sessile form) are more antimicrobial-resistant than planktonic bacteria. Therefore, biofilm-embedded bacteria are more difficult to treat than planktonic types. Consequently, it is essential to detect biofilm-producing bacteria^4^. Antimicrobials' difficulty penetrating biofilms, the appearance of complex drug-resistant bacteria, and biofilm-mediated deactivation or change of antimicrobial enzymes all contribute to antibiotic resistance^5^.

The prevalence of multidrug-resistant bacteria is currently lowering the efficacy of antimicrobial treatments. The advent of these microbes poses a significant threat to healthcare and medicine^6^. So, due to their highly resistant behavior towards conventional antibiotics, there is a huge need for advanced and natural antimicrobial agents that can inhibit biofilm formation early and protect them from antibiotic resistance. In the current scenario, research focuses on developing alternative strategies to combat the pathogenesis of these multidrug-resistant bacteria from natural antimicrobial agents. For the production of a bioactive natural compound, actinobacteria are always the first choice for researchers due to their remarkable quality for valuable secondary metabolite production. Actinobacteria are a treasure trove for identifying numerous secondary metabolites with varied biological functions. Actinobacteria are an advanced category of filamentous bacteria^7,8^.

Antibiotics have been replaced with nanoparticles to tackle MDR-based illnesses. Their nano-formulations can transcend the biological barrier, overcoming the limitations of conventional antibiotic treatments (limited penetration and retention in cells or biofilm)^9,10^. AgNPs are known for their antibacterial efficacy against many pathogenic bacteria^5^. Emerging in recent years, "green synthesis" is an approach that implements nontoxic, naturally occurring herbs to counteract the drawbacks of conventional physiochemical methods. Green synthesized silver nanoparticles use phytochemicals and antioxidants as naturally occurring reducing agents, which is cost-effective and beneficial for mass-scale production. Silver nanoparticle demand has risen significantly in several industries like healthcare, including nano drugs formulation, catalysis, dye reduction, antimicrobial, anticancer, and household goods. Nanomaterials have been utilized as efficient coatings and bactericidal agents to prevent bacterial attachment to surfaces^11,12^. Many studies have examined AgNPs' antibacterial activity; however, the antibiotic mechanisms and associated toxicity are unclear. Silver nanoparticles emit silver ions that have the potential to destroy the bacteria. AgNPs may interact with proteins, lipids, and DNA when they enter microbial cells. AgNPs interact with biological structures or biomolecules to kill microbes. AgNPs are antibacterial because they produce reactive oxygen species (ROS) and free radicals such as hydrogen peroxide, superoxide anion, hydroxyl radical, hypochlorous acid, and singlet oxygen^13^.

The present research aims to evaluate the production of secondary metabolites by *Streptomycetes rochie* LA2(O) and *Microbacterium proteolyticum* LA2(R) to check their antibacterial potential against bacterial meningitis-causing bacteria and their biofilms. Furthermore, advanced microscopic examination and spectrometric analyses were used to characterize the produced AgNPs.

## Results

### Isolation and pretreatment of actinobacteria from soil

As reported in our previous studies, ten soil samples were collected from rhizosphere of medicinal plants from different altitude range of Lucknow, U.P. Based on the antimicrobial screening of isolates; nine isolates were selected. Two strains named *Microbacterium proteolyticum* (MN560041) LA2(R) and *Streptomyces rochei* LA2(O) were confirmed by molecular characterization^14^.

### Morphological identification

#### Gram staining

The powdery white colonies of actinobacteria were isolated from the rhizospheric soil samples, and their morphological characteristics were analyzed. Fourteen isolates were found positive after Gram staining. Gram-positive isolates were chosen for further characterization via microscopic analysis.

#### Endospore staining

Endospores staining of actinobacteria confirmed the presence of both vegetative cells and spore producing cells. Both actinobacterial isolates LA2(R) and LA2(O) were stained and found pinkish vegetative cells and green spores.

#### Scanning electron microscopy (SEM) of actinobacterial isolates

**Figure 1:**
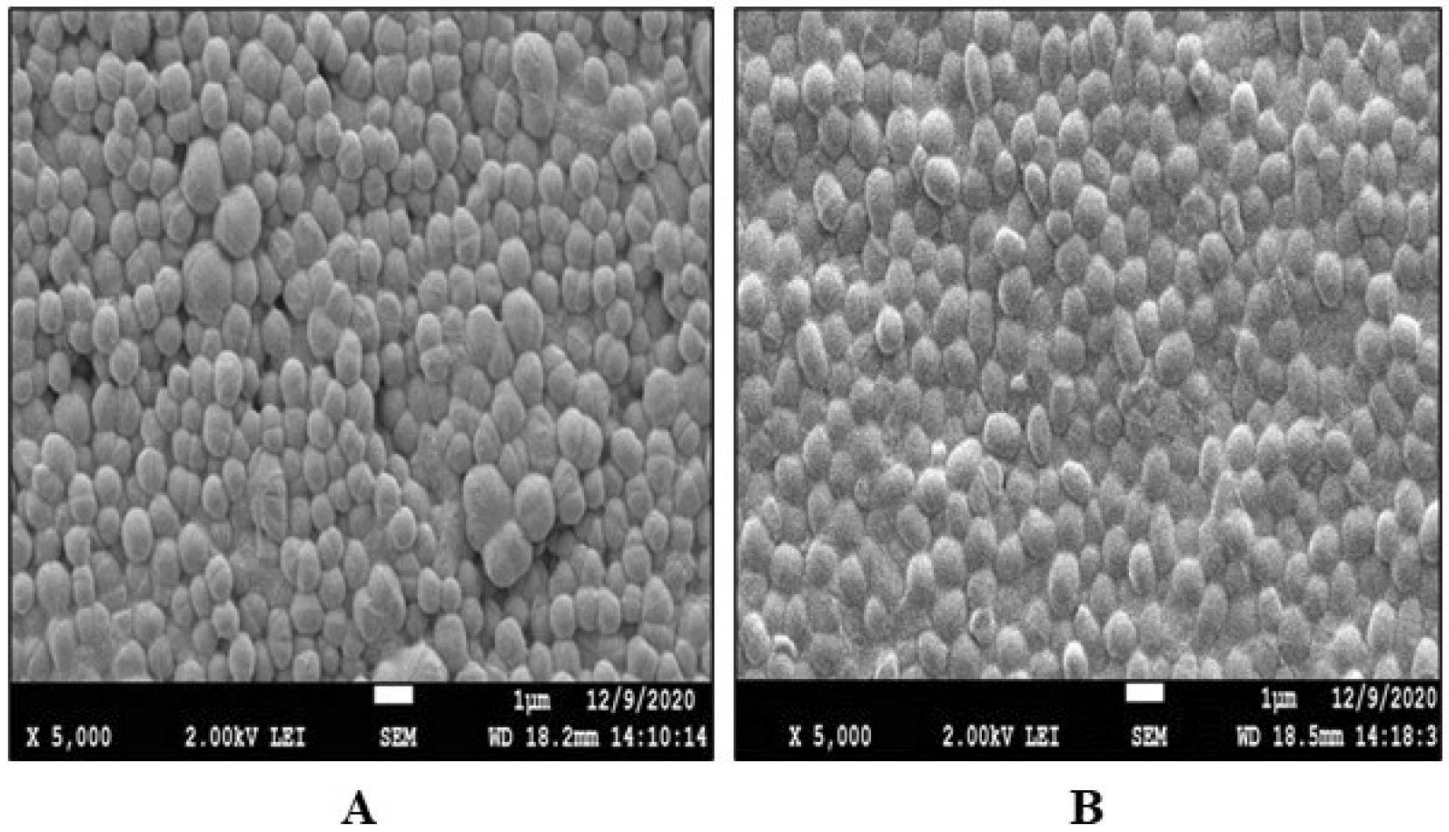
SEM Images of Pure colony of Actinobacteria (**A**) LA2(R) and (**B**) LA2(O) at magnification of 5000 ×.

Objective magnification (5000 ×) was employed to check for spore chains' presence and observe the sporophores' nature in the actinobacteria strains. SEM images of both isolates, i.e., LA2(R) and LA2(O), are shown in Fig. 1A and B, which indicates that morphologically these isolates are spherical in shape of various sizes (15–40 nm) and the average size was calculated to be of 30 nm.

### Molecular analysis of isolated strains

16S rRNA sequence amplification validated the isolates' microscopic appearance and antibacterial activity. Using GenBank's BLAST program, the two most active isolates belonged to Actinobacteria. Phylogenetic analysis confirmed one isolate was *Microbacterium* LA2(R), and another was *Streptomyces* LA2(O). 100% similarity was found for *Streptomyces rochei*. *Microbacterium proteolyticum* was confirmed with 98.62% similarity. Our previous study demonstrates where the partial 16S rRNA sequences of LA2(R) were submitted in NCBI GenBank and got an accession number MN560041^14^.

### Secondary metabolite production and green synthesis of silver nanoparticles

The ISP-2 broth is fermented, and the supernatant is then recovered using ethyl acetate. The solvent phase is removed from the liquid–liquid extract using a rotatory vacuum evaporator, and the remaining crude extract is then combined with methanol and stored. This extracted bioactive compound was then characterized by GC–MS analysis. In our previous research, the n-Hexadecanoic acid was the chief compound with a peak of 14 min retention time (RT) and 95% similarity index^14^.

After 48 h of incubation at 40 °C, 1 mM AgNO_3_ was mixed with 333 g/ml of secondary metabolites LA2(R) and LA2(O). As per the literature reports, the reducing potential generated by secondary metabolites can neutralize AgNO_3_ (aq) into Ag (aq) NPs in water. Evidence of a reaction in a salt-free environment has been confirmed. Similarly, no AgNPs-specific assimilation peak was seen after incubating secondary metabolites in sterile water (s). The surface plasmon resonance (SPR) peak of the AgNPs is responsible for the characteristic absorption maxima at 412 nm for strain LA2(R) (RAgNPs) and 413 nm for strain LA2(O) (OAgNPs).

### Characterization of nanoparticles

#### Color determination

**Figure 2:**
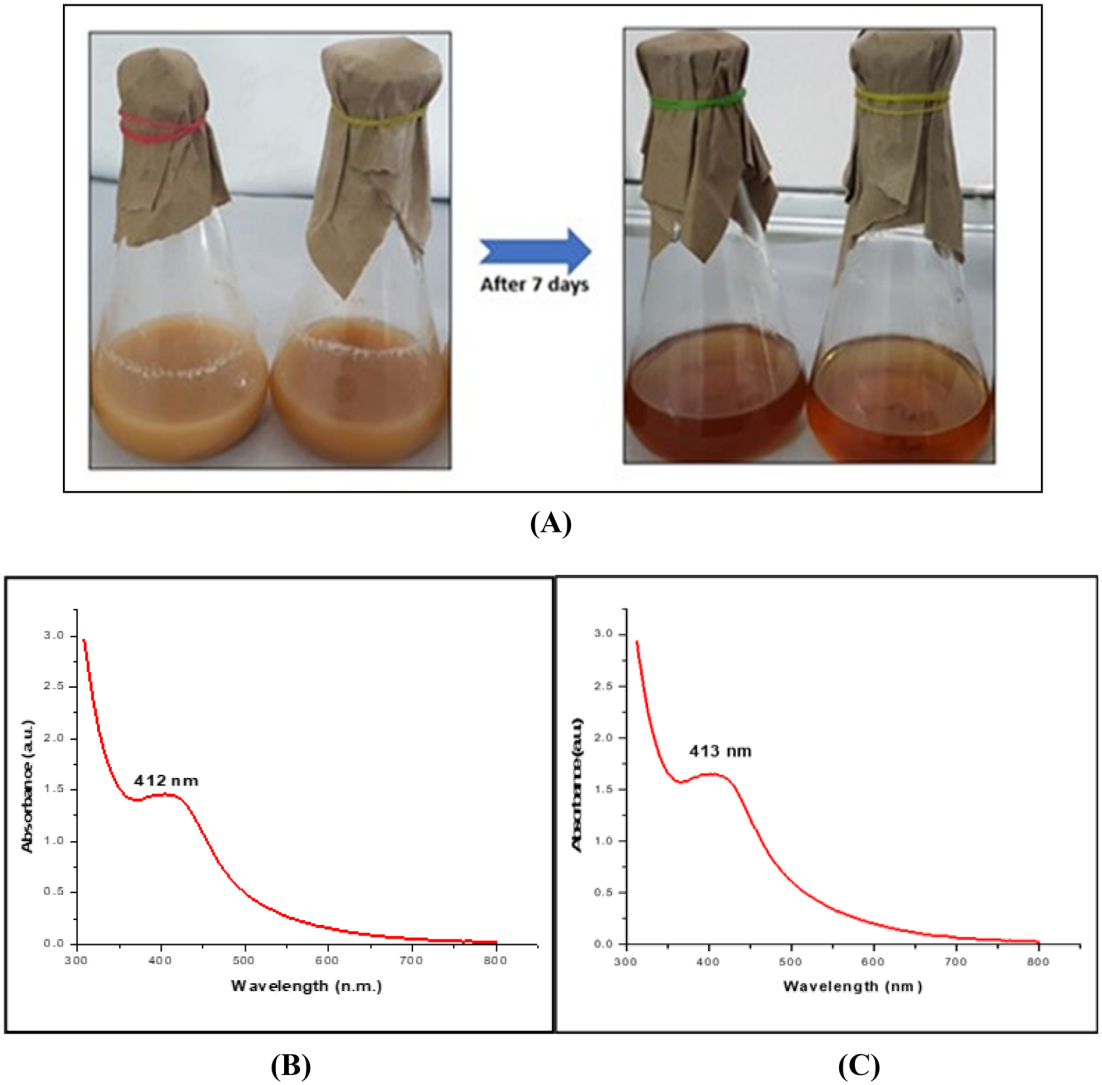
(**A**) Change of color from pale yellow to brownish color shows formation of AgNPs. Characterization of (LA2 R and LA2 O) Silver nanoparticles under UV–Visible Spectroscopy. (**B**) The AgNPs shows a distinct and fairly broad absorption peak centered at 412 nm. (**C**) The AgNPs shows a distinct and fairly broad absorption peak centered at 413 nm.

The first step for the formation of nanoparticle synthesis is the color change. Silver nanoparticle (AgNPs) aggregation to a larger size is marked by a change in color in the solution from yellow to brown (Fig. 2A).

#### UV spectroscopy

A Shimadzu dual-beam Spectrophotometer was used to take UV–Visible spectroscopic readings in a quartz cuvette at a resolution of 1 nm (model UV-1601 PC). The spectra of absorption were taken from 200 to 800 nm. The samples' OD (optical densities) were measured and analyzed through graph^15^. The distinctive peak of 412 nm was detected in the case of LA2(R), and the peak of 413 nm was observed in the case of LA2(O) (Fig. 2B,C).

#### Fourier-transform infrared spectroscopy (FTIR)

**Figure 3:**
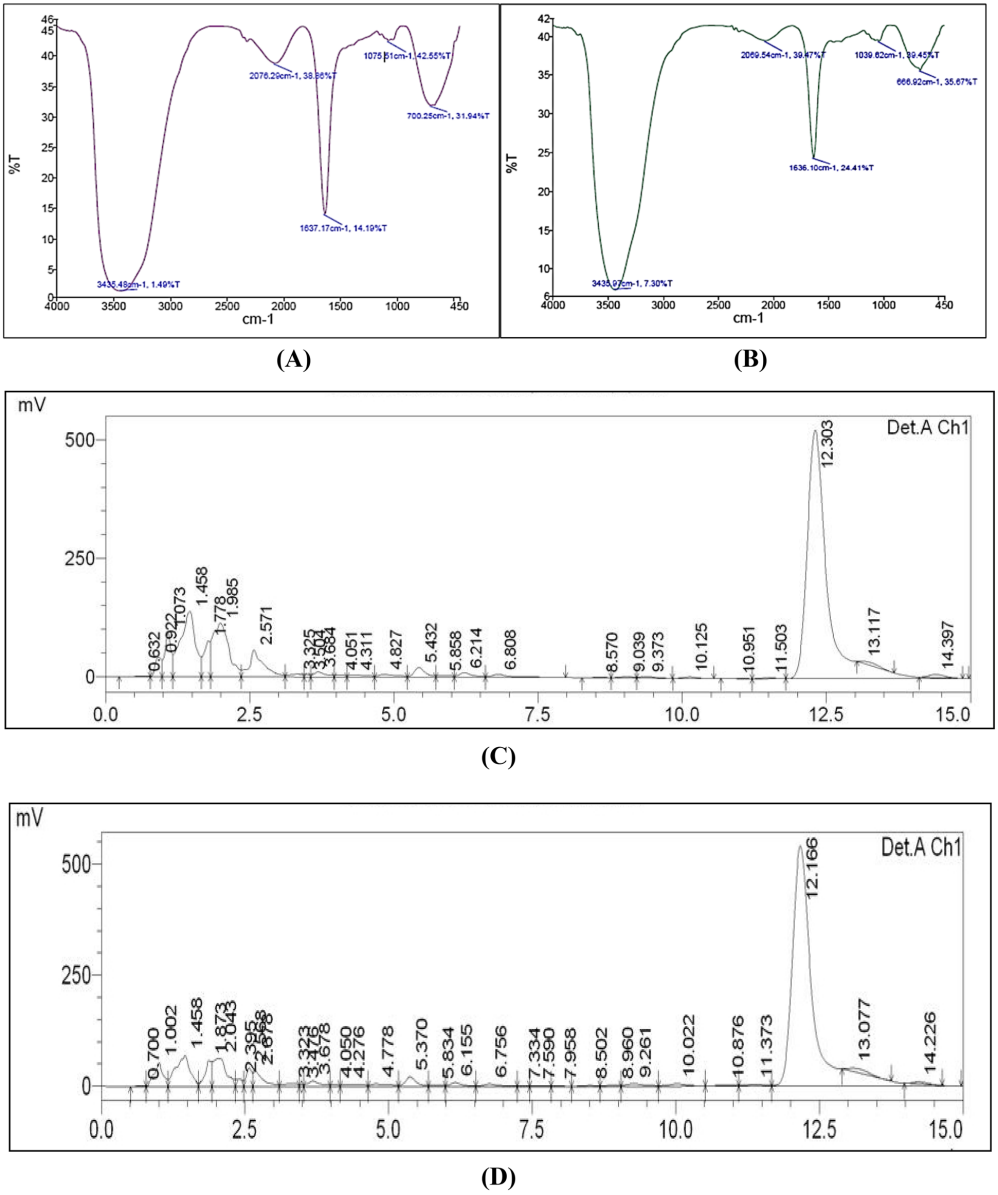
(**A**) Characterization of (LA2 R) synthesized AgNPs by using FTIR Spectroscopy. The FTIR result of the sample (LA2 R) stated that position of Amino (–NH) bond is (3435.48 cm^−1^) with transmittance (1.49%), Alkynes (C≡C cm^−1^) bond is (2076.29) with transmittance (38.86%), Amide (–C=O) bond is (1637.17 cm^−1^) with transmittance (14.19%), Ether (C–O) bond is (1075.51 cm^−1^) with transmittance (42.55%), Alcohol (C–O) bond is (700.25) with transmittance (31.94%). (**B**) The FTIR result of the sample (LA2 O) stated that position of Amino (–NH) bond is (3435.97 cm^−1^) with transmittance (7.3%), Alkynes (C≡C cm^−1^) bond is (2069.54 cm^−1^) with transmittance (39.47%), Amide (–C=O) bond is (1636.1 cm^−1^) with transmittance (24.41%), Ether (C–O) bond is (1039.62 cm^−1^) with transmittance (39.45%), Alcohol (C–O) bond is (666.92 cm^−1^) with transmittance (35.67%). (**C**) HPLC chromatogram of LA2(R) extract. (**D**) HPLC chromatogram of LA2(O) extract.

Infrared analysis based on the Fourier transform was utilized in order to locate the biomolecules that were responsible for the bio reduction of silver ions (Ag^+^) into AgNPs (Ag^0^) as well as the capping of the created Bio-AgNPs. As shown in Fig. 3A and B multiple peaks were observed. FTIR analysis of RAgNPs and OAgNPs revealed the presence of peaks with positions of 1,637.17 cm^1^ and 1636.10 cm^1^ for C=O amide group appearances in the amide I linkage. Amide bond vibrations caused by carboxyl stretching and N–H deformation in proteins containing AgNps are the primary contributors to the development of the amide bands. In addition, the N–H stretching vibration is supported by peaks that appear at 3435.48 and 3435.97 cm^−1^, respectively. In this vibrational phase, it is not affected by the strength of a hydrogen bond and is independent of the conformation of the backbone. Additionally, peaks at 700.25, 666.92 and 1075.51, 1039.62 cm^1^ indicate C–O stretch of the alcohol and ether group, respectively, while the vibration at 2076.29 and 2069.54 cm^−1^ demonstrates C≡C stretch of alkynes. These peaks prove the category of secondary metabolites having the corresponding functional groups of alkynes, and amines which are responsible for the formation of nanoparticles (Fig. 3A,B).

#### High-performance liquid chromatography (HPLC)

HPLC data confirmed the existence of biologically significant chemicals in the aqueous extract by analyzing it using high-performance liquid chromatography (HPLC). Figure 3C and D depicts a typical chromatogram, where we noticed that the highest peak area of 69% and 59% has been covered by the peak at 12.166 and 12.30 retention time, respectively. Rest of the peaks were found to be less than 12%, therefore the compound having higher peak area might be responsible for their activities.

#### Transmission electron microscopy (TEM)

**Figure 4:**
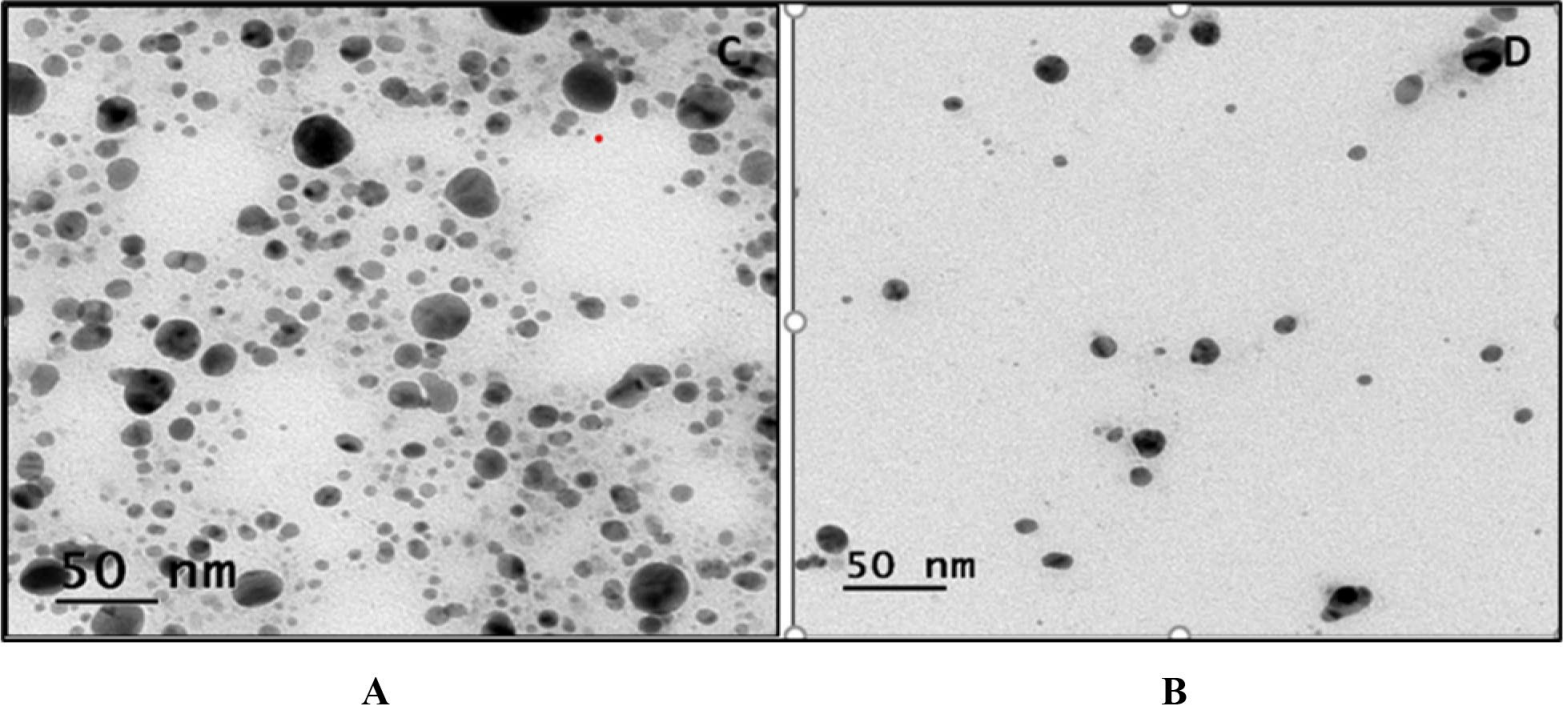
TEM micrograph of AgNPs from supernatant of (**A**) RAgNPs and (**B**) OAgNPs.

One drop of AgNPs solution was used with carbon-coated TEM copper grids to perform TEM, 80 kV Tecnai TM G2 Spirit BioTWIN (FEI Company). TEM image of AgNPs reveals their spherical shape and a diameter ranging from 11 to 42 nm. This finding indicates that the particle was polydispersed, with diameters varying from 11 to 42 nm^16^ (Fig. 4A,B).

### Anti-meningitis activity

**Figure 5:**
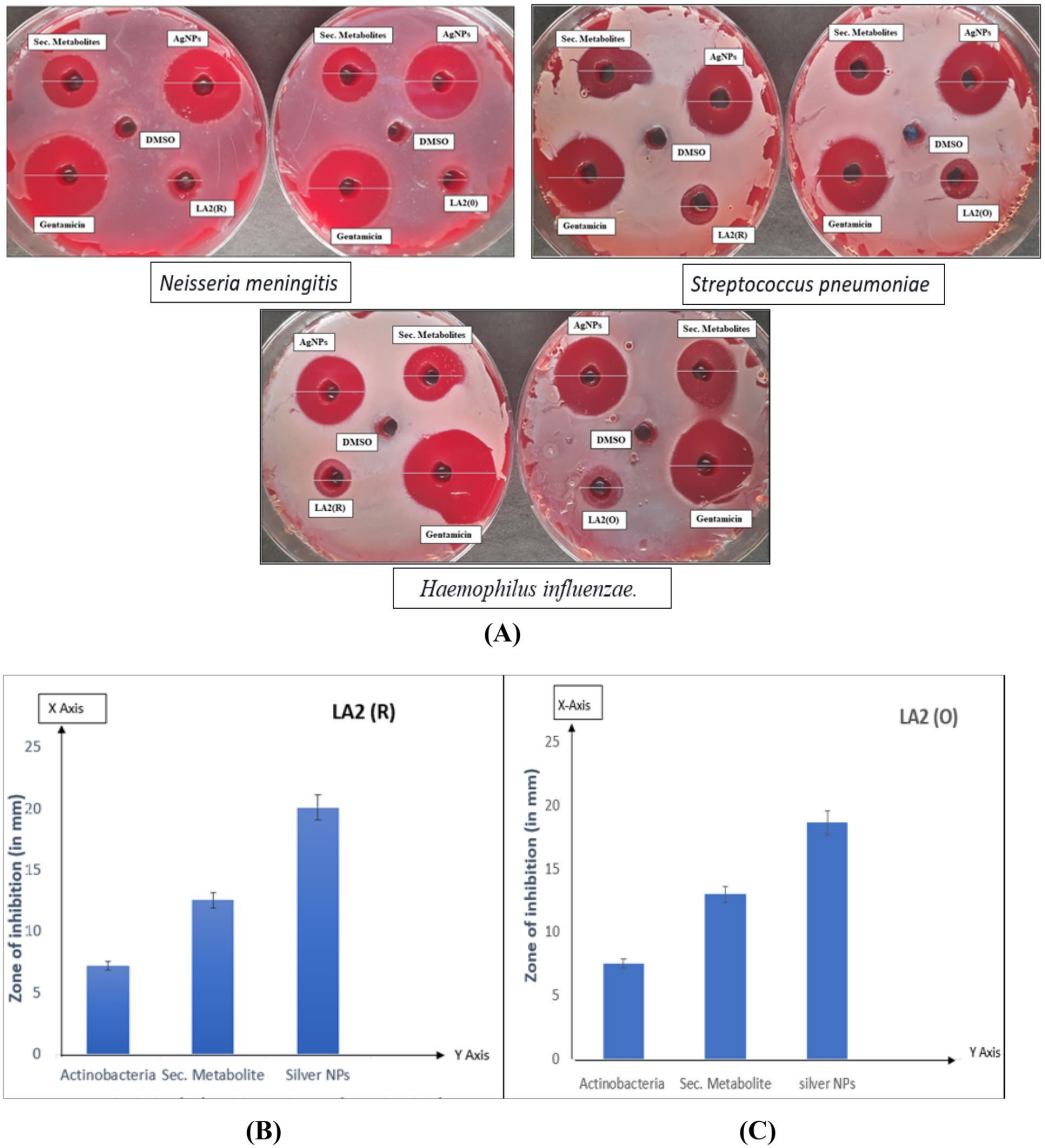
(**A**) Antibacterial activity of Actinobacteria, secondary metabolites and the nanoparticle synthesized actinobacteria against meningitis causing bacteria on Blood Agar. (**B**, **C**) Bar graph showing the comparative analysis of Antibacterial Potential of LA2(R) and LA2(O) Actinobacteria, Secondary metabolites and Silver Nanoparticles of Actinobacteria against bacterial meningitis causing bacteria.

Bacterial meningitis-causing bacteria *Streptococcus pneumoniae* (655)*, Haemophilus influenzae* (3826), and *Neisseria meningitidis* (7981) were procured from MTCC (Microbial Type Culture Collection and Gene Bank), Chandigarh. Silver nanoparticles synthesized bioactive compounds of actinobacteria show better results in inhibiting meningitis-causing bacteria among the comparative analysis of secondary metabolites of actinobacteria and their silver nanoparticles. The pure cultured 24-h growth bacteria were used for the antibacterial activity of meningitis-causing bacteria for the comparative analysis (Fig. 5A). As depicted by the bar graph, following the zone of inhibition, comparison results indicate that silver nanoparticle-synthesized bioactive compounds exhibit excellent effectiveness against meningitis-causing bacteria (Fig. 5B,C). The inhibition activity of these nanoparticles is nearly identical to that of the standard antibiotic, gentamicin. As indicated in Table 1, amongst*. pneumoniae, H. influenzae*, and *N. meningitidis, Haemophilus influenzae* was the most suppressive.

**Table 1:** Zone of inhibition of isolates LA2(R) and LA2(O) against meningitis causing bacteria.

	*Microbacterium proteolyticum* LA2(R) (zone of inhibition in mm)	*Streptomyces rochei* LA2(O) (zone of inhibition in mm)
*Haemophilus influenza* (MTCC No-3826)		
Pure bacteria	9	6
Secondary metabolites	16	14
AgNPs	**32**	29
Drug	34	34
*Streptomyces pnuemoniae* (MTCC No-655)		
Pure bacteria	10	8
Secondary metabolites	19	15
AgNPs	**26**	23
Drug	33	34
*Neisseria Meningitis* (MTCC No-7981)		
Pure bacteria	11	10
Secondary metabolites	17	15
AgNPs	**31**	29
Drug	34	33

### MIC (minimum inhibitory concentration) of nanoparticle synthesize actinobacteria

**Figure 6:**
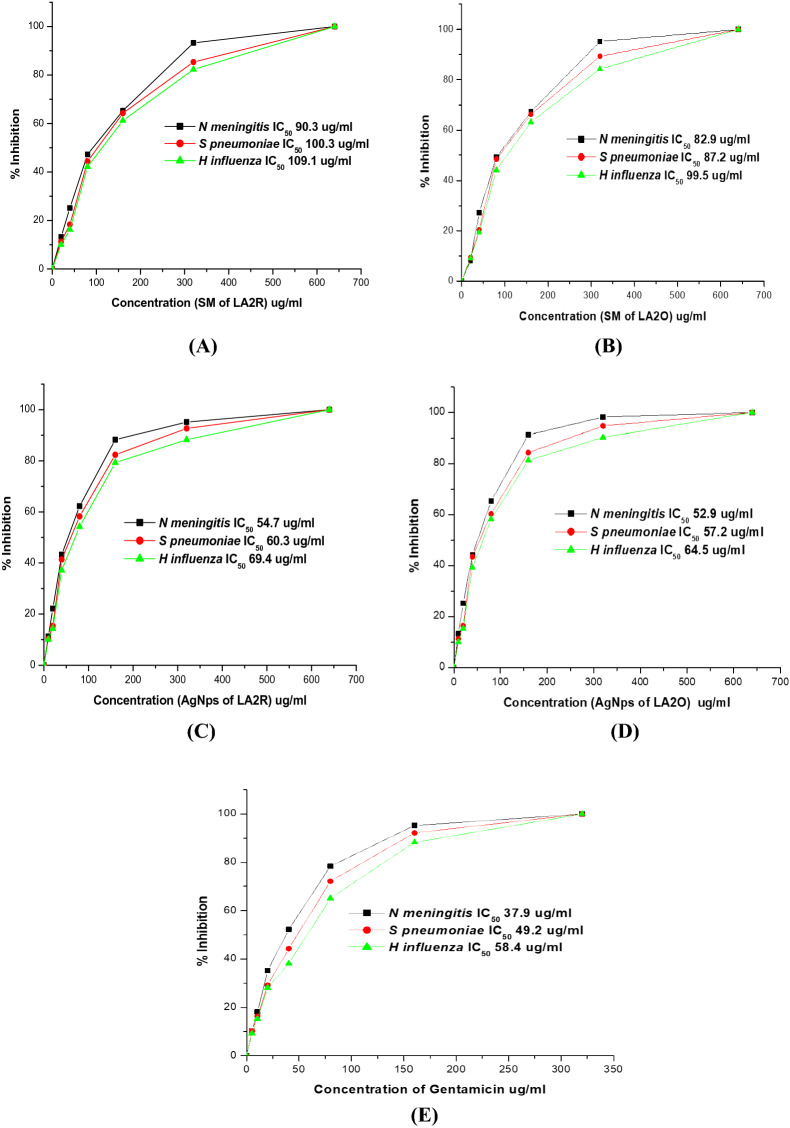
The MIC of (**A**) the secondary metabolite of *Microbacterium* LA2(R), (**B**) the secondary metabolite of *streptomycetes* LA2(O), (**C**) silver nanoparticles of LA2(R), (**D**) silver nanoparticles of LA2(O), and (**E**) The MIC of the antibacterial drug Gentamicin, against meningitis causing bacteria.

Bioactive compounds produced by LA2(R) and LA2(O) isolates were evaluated for their antibacterial efficacy by their minimum inhibitory concentration (MIC). Secondary metabolites from LA2(R) and LA2(O) isolates, as well as silver-produced nanoparticles (RAgNPs and OAgNPs), were tested in 96 well plates to determine the minimal inhibitory concentration against meningitis-causing bacteria. The ELISA plate reader was used to take a reading after 24 h. After incubating the plates at 37 °C for 24 to 48 h, the results were analyzed. The IC50 (half-maximal inhibitory concentration) value of secondary metabolites of LA2(R) was found to be 90.3, 100.3, and 109.1 μg/ml, and the IC50 value of secondary metabolites of LA2(O) was found to be 82.90, 87.20, and 99.50 μg/ml against *N. meningitis*,* S. pnuemoniae*, and *H. influenza*, respectively. Whereas, The IC50 value of silver nanoparticles of LA2(R) was found to be 54.70, 60.30, and 69.40 μg/ml, and the IC50 value of silver nanoparticles of LA2(O) was found to be 52.90, 57.20, and 64.53 μg/ml against *N. meningitis*, *S. pnuemoniae*, and *H. influenza*, respectively. The value of gentamicin`s IC50 was estimated 37.90, 49.20 and 58.40 μg/ml against *N. meningitis*, *S. pnuemoniae*, and *H. influenza,* respectively (Fig. 6).

### Antibiofilm activity of silver nanoparticles

**Figure 7:**
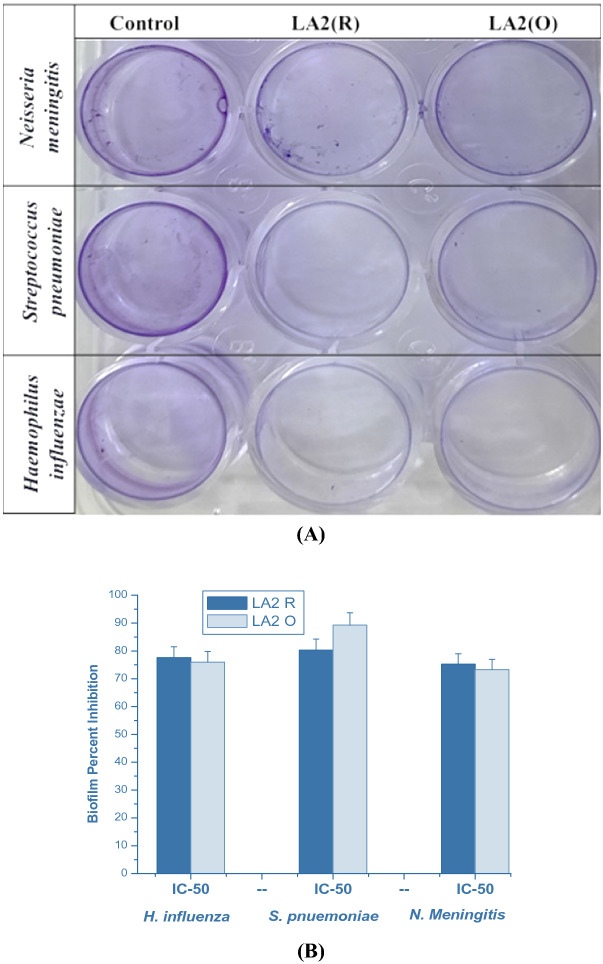
(**A**) Wells depicting visible positive and negative biofilm production lining the wall against the pathogenic strains *H. influenza*, *S. pnuemoniae*, and *N. meningitis*, respectively (**B**) The graphs representing the growth and IC-50 of above specified pathogenic bacteria.

The anti-biofilm activity of the silver nanoparticle of LA2(R) and LA2(O) was explored using the crystal violet staining method. In-microtiter plate wells, test organisms, LA2(R) and LA2(O), were cultivated with and without antibiotics for 24 h for the biofilm formation. LA2(R) has a unique capability to disrupt the biofilms of several pathogenic strains of bacteria. The treatment with IC50 concentration of extract resulted in a considerable reduction (70–80%) in the biofilm formation; it was observed that LA2(R) served as a better antibiofilm agent against meningitis-causing bacteria (Fig. 7A,B).

## Discussion

Meningitis, a potentially fatal systemic infection, has devastating effects in many developing countries due to a lack of financial resources and deteriorating living conditions. To effectively disseminate and administer efficient vaccines to prevent bacterial meningitis worldwide, novel strategies are required. This research has been undertaken in an attempt to produce a medicine that is both successful and practical in the treatment of bacterial meningitis. The actinobacteria *Microbacterium protioliticum* (MN560041) LA2(R) and *Streptomyces rochei* LA2(O) were isolated from the rhizosphere of medicinal plants.

Gram staining was used to characterize the strains morphologically, and gram-positive actinobacterial isolates were chosen for further study. And then, the isolated strains were tested for characteristics like biochemical activity and antibacterial susceptibility. Subsequently, 16S rRNA sequencing was performed by Biokart Pvt. Ltd. in Bangalore, India, for molecular characterization. This data was used in our prior published study^14^. Both vegetative cells and spores were confirmed by endospore staining in the isolates that were first screened. These isolated strains were then viewed under an electron microscope at a magnification of 5000 × to look for spores and determine their form, pattern, and nature^17^.

Secondary metabolites were produced by the actinobacterial isolates *Microbacterium protioliticum* (MN560041) LA2(R) and *Streptomyces rochei* LA2(O), and green synthesis of silver nanoparticles was accomplished by mixing an aqueous solution of 1 mM silver nitrate (50 mL) with supernatant from both actinobacteria isolates. A UV–Vis spectrophotometric analysis was used to analyze the synthesis of AgNPs. As stated by Baker et al. (2021), the color will change to a yellowish-brown. A positive indication was found in the silver nitrate solution treated with actinobacteria supernatant. The distinctive peak of 412 nm was detected in the case of LA2(R), and the peak of 413 nm was observed in the case of LA2(O). In our previous study, we used the well-diffusion method to investigate the antibacterial activity of the resulting LA2(R), LA2(O), and biosynthesized silver nanoparticles against some primary meningitis-causing bacteria obtained from MTCC Chandigarh. The zone of inhibition obtained indicated that the silver nanoparticles had useful antibacterial properties. The FTIR spectrum identified the possible biomolecules responsible for reducing Ag^+^ ions by the cell filtrate.

TEM analysis showed that the particles were spherical and had a diameter ranging from 11 to 42 nm. The shape of the isolates found in this study produced by AgNPs is similar to the results of Abd-Elhady in 2022^16^.

This study and the studies conducted by Tiwari and Mazzetti et al. demonstrate that metabolites derived from actinomycetes can be effective inhibitors of bacteria that cause meningitis *Streptococcus pneumonia*, *Neisseria meningitis,* and *Haemophilus influenza* ^18,19^. Our findings correspond with those made by Tan et al. and Kumar et al., accordingly. In these investigations, neonates with bacterial meningitis with high CSF protein content had a poor prognosis^20,21^.

The use of silver nanoparticle synthesis has been shown to increase the activity of Actinobacteria^22^. To overcome multi-drug resistance and biofilm-based infections, nanoparticles have been proposed as a potential alternative to replace antibiotics. Park et al. hypothesized that biosorption is liable for biofilm deactivation, implying that LA2(R) could be utilized as a biofilm-disrupting agent. Since typical antibiotic treatments are less effective in cells or biofilms because of their lower penetration and retention, researchers turned to nanoformulations, which have the potential to both enter and remain within cells. Nanomaterials have been widely employed as effective coatings to prevent bacterial attachment to surfaces and bactericidal agents in medical applications. Our study, like several others, found silver nanoparticles (AgNPs) to have anti-microbial action against a wide range of pathogenic microorganisms^23^. When tested against the medicine employed as a control, the metabolites derived from actinomycetes coated with AgNPs demonstrated much greater bactericidal potency in a concentration-dependent manner.

## Material and methods

### Media and bacterial strain

This study only employed analytical research-grade substances. All chemicals and media were procured from Hi-Media Laboratories (Mumbai, India). The glassware was washed using 6N HCl and K_2_Cr_2_O_7_, rinsed with sterile water and then dried in a hot air oven. The pathogenic, bacterial meningitis-causing strains, i.e., *Streptococcus pneumoniae* (MTCC-655), *Haemophilus influenzae* (MTCC-3826), and *Neisseria meningitis* (ATCC 13090-0454L) were procured from MTCC Chandigarh, India.

### Isolation of actinobacteria from soil

Ten soil samples were collected from the rhizospheres of medicinal plants, including *Asparagus racemosus, Withania somnifera, Salvia officinalis, Rouwolfia serprntina*, and *Ocimum sanctum*, and stored in sterile bags at 4 °C^24^. All soil samples were dried in the air and processed with calcium carbonate before isolation to prevent the entry of other unwanted bacteria and microbes^17^. Up to tenfold serial dilutions of treated samples were made. Then 0.1 ml aliquots were spread across various selective media, including Actinomycetes Isolation Agar (AIA), Glycerol Asparagine Agar (GAA), Yeast Malt Agar (YM Agar) (ISP Medium No. 2, HiMedia), Starch Casein Agar (SCA) and Bennet’s agar, along with the antibacterial drug, i.e., cycloheximide (50 mg/ml) and antifungal medication, i.e., nystatin (50 mg/ml), and incubated at 28 °C for seven to ten days^14^. According to Bergey's Manual, actinobacteria were identified using macroscopic and microscopic examinations and physiological testing^25^.

### Morphological identification

After inoculating the bacteria onto Actinomycetes Isolation Agar (AIA) media, actinobacteria were isolated as powdery white colonies, and their morphological properties were examined. Under a microscope, the most prevalent bacteria (later classified as Actinobacteria) appeared to be powdery white colonies with chains of spores and branching filaments that contained both ariel and substrate mycelia. The shape of the spore chains produced by the growing isolates was examined through endospore staining.

#### Gram staining

Actinobacteria are usually gram-positive bacteria, so gram staining was performed for the screening of gram-negative bacteria. Bacteria are positively identified as actinobacteria due to their characteristic filamentous structure^26^. The presence of spores on their structures prompted the use of endospore techniques to verify this finding.

#### Endospore staining

Bacterial endospores have been carefully investigated for many years due to their significance in commercial and medicinal industries. Actinomycetales contain the most spore-forming bacterial taxa and the greatest variety of spore forms. Actinomycete spore research may contribute to our understanding of spore qualities in general. The discovery of light green spores and a red vegetative cell color revealed the most remarkable distinctions between vegetative cells and spores. Malachite green staining (Schaeffer-Fulton method) is the most frequent staining technique for endospores. Malachite green can also be used as a simple stain for bacterial cells. The malachite green (primary dye) is flooded into the endospore using heat in the Schaeffer-Fulton procedure. Washed with distilled water decolorizes the cell, rendering the Endospores pale green and the Vegetative cells brownish-red to pink^27^.

#### SEM

SEM (Scanning electron microscope) was used to examine the isolate’s detailed morphologies. The isolates *Microbacterium protioliticum* (MN560041) LA2(R) and *Streptomyces rochei* LA2(O) were examined under a microscope at a magnification of 5000 × to check for the presence of spores, their shape, pattern, and nature^17^. The sample was prepared as the protocol described by Siddharth et al.^28^. Sterilized aluminum stubs and coverslips were inserted at 45 °C for SEM analysis. Isolates were injected along the stub's agar line and cultivated at 28 ± 2 °C for seven days. The stubs were removed, plated with gold under vacuum for 25–30 min, and viewed with a scanning electron microscope (JEOL JSM 7610f) from BSIP, Lucknow^28^.

### Secondary metabolite production and green synthesis of silver nanoparticles

Secondary metabolites of isolates *Microbacterium protioliticum* (MN560041) LA2(R) and *Streptomyces rochei* LA2(O) were produced by Submerged State Fermentation (SmF) defined by Salim et al. (2017). The isolates were inoculated in ISP-2 medium (4.0 g of yeast extract; 10.0 g of malt extract; 4.0 g of dextrose; 20.0 g of Agar; 1L of distilled water, pH 7.3) and incubated in a rotary shaker at 200 rpm at ± 28 °C for 5 to 7 days. Both culture supernatants were employed in subsequent tests after being centrifuged at 12,000 rpm^29^.

Green synthesis of AgNPs was performed using 1 mM silver nitrate aqueous solution (50 μl) mixed with supernatant of both actinobacterial isolates (50 ml), and the pH was adjusted to 8.5. One control experiment was also performed without inoculation of supernatant. After 48–72 h of incubation, in the presence of light, the color of the broth changes from yellow to dark brown. The mixture of flasks was labeled and incubated at 37 °C in a rotary shaker at 200 rpm for 5 to 7 days. UV–Vis spectrophotometer monitored the synthesis of AgNPs.

### Characterizations of AgNPs

The presence of extracellular silver nanoparticle synthesis was confirmed with color change, UV–visible spectrophotometer, Fourier-transform infrared spectroscopy analysis, Transmission electron microscopy, and High-Performance Liquid Chromatography.

#### Color determination

The first step for the establishment of nanoparticle synthesis is a color change. Silver synthesized NPs combination to a bigger size is noticeable by the color change from yellow to brown^30^. The existence of dark brown color may result from the AgNO_3_ being reduced and the surface plasmon resonance (SPR) action being activated^31^.

#### UV spectrometry

UV–Vis spectrometry is one of the most effective and convenient approaches for confirming nanoparticle production. Absorption measurements made using UV–visible spectrometer were used to calculate the metal ion reduction^32^. The characterization of nanoparticles considers light wavelengths between 200 and 800 nm. Using a UV–Vis spectrophotometer and sterile water as a reference, the absorbance spectrum of the colloidal sample was determined in the 200–800 nm rangema^10^. The UV–visible spectroscopy was measured in a quartz cuvette using a 1 nm resolution Shimadzu dual-beam spectrophotometer (model UV-1601 PC). Visualizations of the samples' measured optical densities (O.D.) were analyzed^15,31^.

#### FTIR

FTIR assay was used to estimate the functional groups on the NP's surface. NPs were centrifuged at 5000 rpm for 20 min, the pellets were washed several times, dried, and the powder was encapsulated with KBR to be examined using FTIR at 400–4000 cm^−1^. FTIR delivers information about the interaction of proteins and nanoparticles. The purpose of FTIR is to analyze how the sample absorbs much light at each wavelength^23,33^.

#### TEM

TEM (Transmission-Electron-Microscopy) was accomplished by drying a drop of GNP solution on carbon-coated TEM copper grids. The inorganic core percentage was determined by drying a drop of produced silver nanoparticle (suspension) on a carbon-coated TEM copper grid at 80 kV using a TEM Tecnai TM G2 Spirit Bio-TWIN (FEI, Hillsboro, OR, USA)^16^.

#### High-performance liquid chromatography (HPLC)

HPLC analyzed the aqueous extract to verify the presence of biologically active chemicals that caused the reduction of silver ions. To accomplish this, an Agilent 1260 system was used. The extract was filtered twice and then poured into an HPLC vial to be eluted with a solvent mixture of methanol, acetic acid, and deionized water. The solvent mixtures were given the letters A (10: 2: 88, v/v) and B (90: 2: 8, v/v). The chemicals were discovered by comparing sample results to reference materials and industry standards^34^.

### Anti-meningitis activity

#### Antagonistic activity of actinobacteria

Isolated strains of actinobacteria have demonstrated high antagonistic activity against *N. meningitidis, H. influenzae*, and *S. pneumoniae* through agar well diffusion method. In this method, the microbial inoculum is dispersed throughout the agar plate surface. A 6–8 mm hole is drilled aseptically with a sterile cork borer, and 20–100 μL of the antimicrobial compound at the needed concentration is poured into the well, followed by incubation of agar plates at 28–30 °C for 24–48 h. The antibiotic agent spreads throughout the agar media, inhibiting the bacterium strain from proliferating^35^. Using a ruler, the growth inhibition zones surrounding the two wells for each medication concentration are measured in millimeters. The growth-inhibitory zone of the extract concentrations for each of the microorganisms was estimated using the mean of the duplicate trials for each concentration of the extracts^36^.

#### Antibacterial susceptibility testing of secondary metabolites and silver nanoparticles of actinobacteria

The antibacterial activity of silver nanoparticles was examined with the help of the well diffusion method. 100 μl secondary metabolites (1 mg/ml) produced by both strains of actinobacteria, i.e., LA2(O) and LA2(R), were poured into wells to check their antibacterial activity. And 100 μl of RAgNPs and OAgNPs silver nanoparticles (100 µg/ml) concentration were poured into other wells. After the spreading of the above-mentioned meningitis-causing bacteria. The antimicrobial efficacy of actinobacterial secondary metabolites and their AgNPs shows the remarkable inhibition of bacterial meningitis-causing bacteria.

#### Minimum inhibitory concentration (MIC) of actinobacterial silver nanoparticles and their IC-50

Microdilution was used to determine the MIC values in 96-microwell plates with triplicates. The MIC was the minimum concentration of antimicrobial extract that inhibits the observable growth of a microorganism under the test following incubation. The minimum inhibitory concentration of secondary metabolites of isolates LA2(R) and LA2(O) and their silver synthesized nanoparticles, i.e., RAgNPs and OAgNPs, were tested against meningitis-causing bacteria. A pure culture of a single microbe was cultivated in broth to determine the MIC. The antibacterial agent was diluted in a sterile diluent in a ratio of 1:1. The inoculated and serially diluted antimicrobial compound was incubated for a fixed period at the appropriate temperature for the test organism. To check for microbial growth after incubation, a series of dilution vessels were inspected for turbidity and a pellet of microorganisms in the bottom of every vessel. The last tube in the dilution series that indicates no growth refers to the antimicrobial agent's MIC concentration. Gentamicin antibiotic was used as a control in this study^15^.

The IC50 values of the silver nanoparticles produced by LA2 and its active secondary metabolites (R and O) were calculated using the same methodology. The smallest percentage of actinobacterial secondary metabolites was designated as the IC50 value of LA2 (R and O) and their silver nanoparticles, at which the growth of microorganisms was inhibited by 50%.

#### Antibiofilm activity

The National Institute of Health (NIH) and the Centers for Disease Control and Prevention indicate that between 65 and 80% of all illnesses are caused by biofilm-forming microbes^25^. LA2(R) has found the remarkable ability to destroy the biofilms of many harmful bacterial species. The biofilms of *N. meningitidis, H. influenzae,* and *S. pneumonia* were produced using the method reported by Kemung et al., with minor alterations^37^. Here in this method, the biofilm production was investigated using the traditional crystal violet staining method. By employing a crystal violet colorimetric assay, we were able to determine the viability of biofilms definitively. This inhibitory activity of LA2(R) and LA2(O)may be elucidated by several reasons, including the effectiveness of the antimicrobial activity and the other chemical properties, such as the empathy between materials and biofilms^38^. The percentage (%) attachment in the present study was calculated by using the following equation:

$$Attachment\%= \frac{Absorbance \, of \, sample}{Absorbance \, of \, control}\times 100$$

The percentage of biofilm inhibition was determined using the following equation:

$$Biofilm\; \% \; Inhibition=\left[control \, OD 490 \, nm-test \, OD \, 490 \, nm\right]\times 100$$

### Analytical statistics

The average and standard deviation of three different experiments were given as the results. As stated in a previous study, the statistical analysis was performed using the American Origin 6.0 tool^39^. All experiments were conducted in triplicate and repeated twice, and the data here are presented as mean ± SD.

## Conclusion

Our results indicated that *Microbacterium proteolyticum* LA2(R) and *Streptomyces rochei* LA2(O) isolates could efficiently synthesize bioactive AgNPs using an inexpensive, eco-friendly, and non-toxic biological method. UV–Vis spectrophotometry, TEM, FTIR, and HPLC studies confirmed the synthesis. The silver nanoparticles of actinobacteria, i.e., RAgNPs and OAgNPs can be used as a potent antibacterial agent for bacterial meningitis, causing bacteria to counter the traditional drugs for meningitis. The Biofilms produced by *S. pneumoniae, H. influenzae,* and *N. meningitidis* were also degraded by the nanoparticles of RAgNPs and OAgNPs by 73.14%, 71.89% and 64.81%, respectively. Isolate *Microbacterium proteolyticum* LA2(R) showed better results in comparison to *Streptomyces rochei* LA2(O), as LA2(R) has better antibacterial activity, and their synthesized nanoparticle shows the maximum zone of inhibition in well diffusion method against meningitis causing bacteria and against their biofilms. Our findings provide insight into developing new antimicrobial agents with the synergistic enhancement of the antibacterial mechanism against pathogenic meningitis-causing bacteria.

## Data Availability

All data generated or analyzed during this study are included in this published article.
